# 3D Arrays of Super-Hydrophobic Microtubes from Polypore Mushrooms as Naturally-Derived Systems for Oil Absorption

**DOI:** 10.3390/ma12010132

**Published:** 2019-01-03

**Authors:** Gianluca Balzamo, Naval Singh, Ningjing Wang, Goran T. Vladisavljević, Guido Bolognesi, Elisa Mele

**Affiliations:** 1 Materials Department, Loughborough University, Loughborough LE11 3TU, UK; G.Balzamo@lboro.ac.uk (G.B.); n.wang3-16@student.lboro.ac.uk (N.W.); 2 Chemical Engineering Department, Loughborough University, Loughborough LE11 3TU, UK; N.Singh@lboro.ac.uk (N.S.); G.Vladisavljevic@lboro.ac.uk (G.T.V.); G.Bolognesi@lboro.ac.uk (G.B.)

**Keywords:** biomimetics materials, porosity, oil/water separation, micro-tubes

## Abstract

Porous materials derived from natural resources, such as Luffa sponges, pomelo peel and jute fibres, have recently emerged as oil adsorbents for water purification, due to their suitability, low environmental impact, biodegradability and low cost. Here we show, for the first time, that the porosity of the fruiting body of polypore mushrooms can be used to absorb oils and organic solvents while repelling water. We engineered the surface properties of *Ganoderma applanatum* fungi, of which the fruiting body consists of a regular array of long capillaries embedded in a fibrous matrix, with paraffin wax, octadecyltrichlorosilane (OTS) and trichloro(1H,1H,2H,2H-perfluorooctyl)silane. Morphological and wettability analyses of the modified fungus revealed that the OTS treatment was effective in preserving the 3D porosity of the natural material, inducing super-hydrophobicity (water contact angle higher than 150°) and improving oil sorption capacity (1.8–3.1 g/g). The treated fungus was also inserted into fluidic networks as a filtration element, and its ability to separate water from chloroform was demonstrated.

## 1. Introduction

Porous materials with special wettability (super-hydrophobicity and lipophilicity) find application as membranes and sponges for water and wastewater treatment [1,2,3,4,5,6]. They combine engineered wetting properties (water contact angle higher than 150°, sliding angle lower than 10°, and low oil contact angle) and interconnected porosity to repel water while promoting the flow of oily substances and other pollutants through the pores. 

In recent years, oil adsorbents based on natural porous materials have emerged as potential candidates for oil-water separation because they are abundant, sustainable, biodegradable and inexpensive [7]. Recent papers have reported on the super-hydrophobic treatments of natural Luffa sponges (*Luffa cylindrica*). They are formed of a three-dimensional (3D) network of cellulose-based microfibres [8]. Polyhedral oligomeric silsesquioxane [9], fluorinated silica nanoparticles [10] and waterborne polyurea adhesives containing silica nanoparticles [11] have been used to functionalise Luffa sponges. The treated sponges were characterised by a water contact angle (WCA) between 152° and 156°, and an oil absorption capacity (diesel, vegetable oil, chloroform and toluene) in the range of 3–10 g/g. In another study, the porous, honeycomb-like internal structure of pomelo peel (*Citrus maxima*) was modified with acetic anhydride and styrene to enhance the sorption of diesel and lubricating oil [12]. Untreated pomelo peel exhibited a WCA of 82° and sorption capacity of 6 and 9 g/g for diesel and lubricating oil, respectively. Modified peels showed an increased WCA (up to 128°) and a maximum oil sorption capacity of 19 and 26 g/g for diesel and lubricating oil, respectively. The regular micro-porosity of jute fibres has also been used to create oil sorbents [13]. The jute fibres were functionalised with silica via the sol-gel method and subsequently made hydrophobic (WCA of 136°) with octadecyltrichlorosilane (OTS). The maximum adsorption capacity of the treated fibres was 8 g/g for diesel. In another study, silk fibres were modified with an environmentally friendly enzyme-etching approach and then functionalised with methyltrichlorosilane to achieve super-hydrophobicity (WCA between 140° and 160°) and oil-water separation [14].

**Figure 1:**
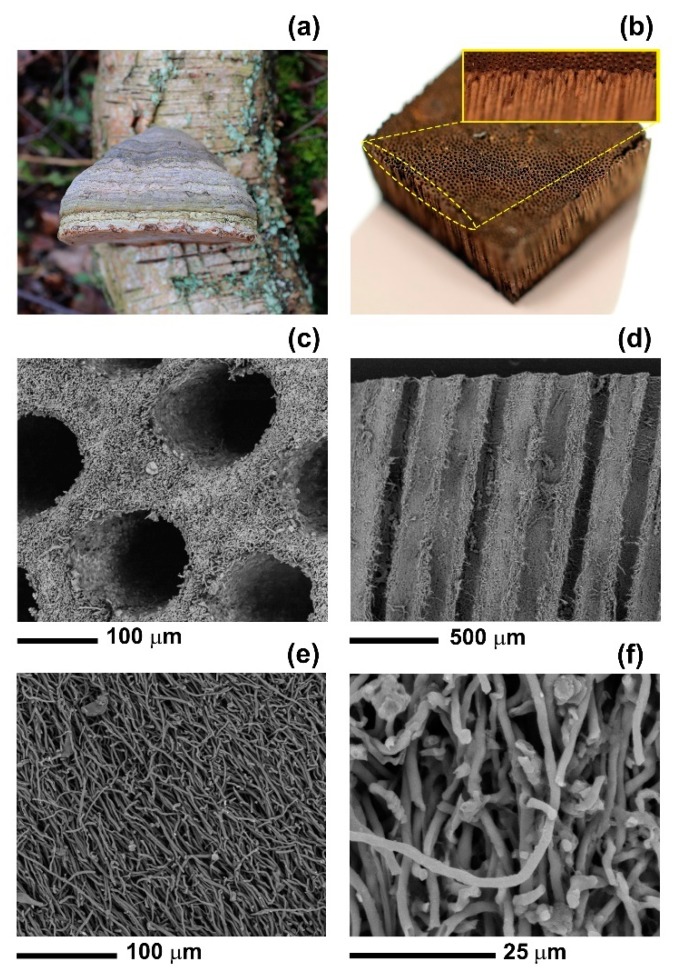
(**a**) Photograph of a *Ganoderma* growing on a fallen birch in South Yorkshire (UK). (**b**) Photograph of a sample of the spore-bearing fruiting body of a *G. applanatum* mushroom. Inset: high magnification photograph of the fungus microtubes. SEM images of (**c**) the bottom surface and (**d**) the cross section of the fruit body of *G. applanatum*. (**e**) and (**f**) SEM images at different magnification of the fibrous walls of the microtubes.

Here, we study how the 3D porous structure of the fruiting body of polypore fungi (also known as bracket fungi) of the genus *Ganoderma* can be functionalised to achieve super-hydrophobicity and enhanced oil sorption. The *Ganoderma* genus comprises of mushrooms that grow on decaying logs or tree stumps (Figure 1a) and are widely studied for their bioactive polysaccharides and triterpenoid compounds that have antioxidant, antitumor and antibacterial properties [15,16,17]. So far, macro- and microscopic features of these fungi have been primarily investigated for taxonomic purposes [18,19]. In this work, we show, for the first time, that the unique porous structure of the fruiting body of *Ganoderma* is advantageous to create systems with engineered water and oil absorption capacity. We selected *Ganoderma applanatum* for its porosity that is characterised by tubular long pores embedded in a fibrous matrix. We treated the fungus with paraffin wax (PFW), OTS or trichloro(1H,1H,2H,2H-perfluorooctyl)silane (FOTS) to achieve high WCA (higher than 150° in some cases) and high oil sorption. We demonstrated that the modified fungus can be used as a recyclable oil sorbent and can be embedded in plastic tubes to create a filter media for oil-water separation.

## 2. Materials and Methods

### 2.1. Materials

Paraffin wax (PFW) (melting point ≥ 65 °C), toluene (anhydrous, 99.8%), trichloro(1H,1H,2H,2H-perfluorooctyl)silane (FOTS) (97%), trichloro(octadecyl)silane (OTS) (≥90%), hexane (95%), acetone (≥99.9%), ethanol (≥99.8%), chloroform (≥99.5%), silicone oil and dioxane (anhydrous, 99.8%) were purchased from Sigma Aldrich (Gillingham, UK). Natural blue colour food dye and rapeseed oil were purchased from a local market. Water with analytical grade purity was purchased from Fisher (Loughborough, UK).

### 2.2. Functionalisation of Ganoderma Applanatum Fungi

The fruiting body of *Ganoderma (G.) applanatum* fungi was cut into samples of around 1 cm^3^. The samples were soaked and washed with warm water (approximately 65 °C) multiple times to remove dirt particles and soluble impurities. The samples were then dried out overnight in a fume cupboard. Four different treatments were conducted separately to change the wetting properties of the dried samples. 

Treatment 1: PFW (5% *w*/*v* (weight/volume)) was dissolved in Toluene at 90 °C for 60 min under stirring until a clear solution was obtained. The fungi samples were then immersed in warm PFW/toluene homogeneous solution (approximately 70 °C) for 30, 60 and 120 s. The PFW dip-coated samples were dried under a fume hood for 1 h and then in an air oven at 50 °C for 4 h.

Treatment 2: A droplet of FOTS was deposited on a glass slide and placed inside a plastic vacuum desiccator, together with the dried *G. applanatum* samples. The samples were left for 2 h under vacuum, to allow the functionalisation of the samples through evaporation of FOTS.

Treatment 3: FOTS was mixed with ethanol (8% *v*/*v* (volume/volume)) for 1 h at room temperature under stirring. The samples were then immersed in the FOTS/ethanol solution for 4 h. The FOTS dip-coated samples were then dried for 3 h in an air oven at 70 °C.

Treatment 4: OTS was dissolved in hexane (4% *v*/*v*) for 5 min at room temperature under stirring. The samples were soaked in the OTS/hexane solution for 5 min and then thoroughly washed with hexane. The resulting OTS dip-coated samples were dried out in an oven at 70 °C for 1 h. 

### 2.3. Sample Characterisation

Static water contact angle (WCA) and oil contact angle (OCA) of treated *G. applanatum* samples were measured using a DataPhysics OCA 20 instrument (Filderstadt, Germany), using 5 μL droplets at ambient temperature. An average of 10 different zones per sample were analysed. Advancing and receding angles were measured by increasing and reducing the volume of a 5 μL water droplet. Images were recorded and evaluated every 1 µL.

The morphology of the pristine and treated *G. applanatum* sample surfaces was analysed using the Scanning Electron Microscope (SEM) Hitachi TM3030 (Tokyo, Japan) using an acceleration voltage of 15 kV. Prior to transferring the samples into the SEM chamber, a conductive layer of 90s gold/palladium (Au/Pd) was deposited onto the sample surface using a Quorum Q150T ES sputter (Laughton, UK).

The X-ray diffraction (XRD) analysis was carried out by a Bruker D2 PHASER (Billerica, MA, USA) desktop diffractometer with a rotating copper anode CuKα (wavelength λ = 0.1542 nm) in the transmission mode (30 kV, 10 mA). A 2*θ* angle in the range of 5–40° was scanned at a speed of 1 °/s.

The maximum oil sorption capacity of the treated samples was evaluated via immersion into the oils and organic solvents for 5 min. Then, the samples were suspended for 30 s to remove the excess of oil and organic solvent, and weighed on a digital balance. The weights of the samples before and after oil absorption were used to calculate the maximum sorption capacity according to the following formula:

$$Q\left(\%\right)=\frac{m_{w}-m_{d}}{m_{d}}\times 100\%$$

where Q(%) is the sorption capability, $m_{d}$ and $m_{w}$ are the weights of samples before (dry sample) and after (wet sample) the oil sorption, respectively. 

The recyclability and possibility of reusing the OTS-modified *G. applanatum* samples for sorption of organic liquids was also investigated. Three samples were immersed in dioxane for 5 min and, after removing the oil excess, weighted to estimate the maximum sorption capacity. Then, the samples were washed in ethanol and dried overnight in a desiccator at 50 °C. This procedure was repeated 3 times.

The abrasion resistance of the treated samples was evaluated by a multistep dry manual test. The sample surface was abraded firmly with a gloved hand (300 mm nitrile glove) using a back and forth movement for 50 times [20]. The wetting properties of the abraded samples were then analysed.

Statistical analysis was performed on independent trials, which were repeated at least three times. Mean and standard deviation (SD) were calculated. One-way analysis of variance, followed by Tukey’s multiple comparison test, with a confidence interval of 95% for mean, was used to analyse the results by Minitab software (*p* < 0.05).

## 3. Results and Discussion

The fruiting bodies of *G. applanatum* consisted of a large number (30–40 pores/mm^2^) of long (up to 20 mm [21,22]) and regular tubes (Figure 1b). The apertures of the tubes from the underside of the fungus had a circular to ovoid shape with a diameter ranging between 80 to 100 µm (Figure 1c,d). The walls of the tubes consisted of a network of microfibres with an average diameter of 2 µm (Figure 1e,f). These tubes are used by the fungi to eject their basidiospores. 

**Figure 2:**
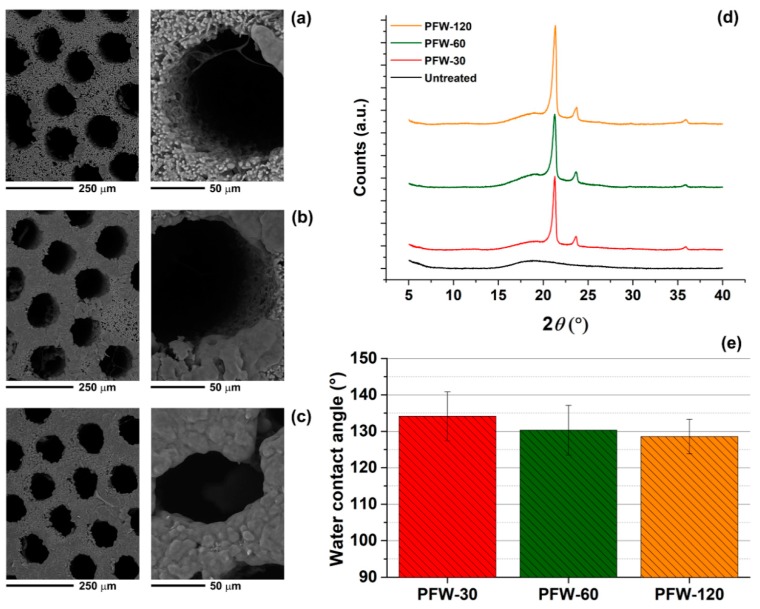
(**a**–**c**) SEM images, at two different magnifications, of the surface porosity of *G. applanatum* samples treated with paraffin wax for 30, 60 and 120 s, respectively. (**d**) XRD patterns of untreated and paraffin wax (PFW)-treated samples. (**e**) Water contact angle analysis of PFW-30, PFW-60 and PFW-120 samples.

The 3D porous structure of fungus was naturally hydrophilic and absorbed liquids. This was also due to the capillary action exerted by the tubular pores. We performed three different surface treatments of the *G. applanatum* samples to control their wetting properties. The first type of treatment consisted of dip-coating the samples in a paraffin wax/toluene solution for 30 s (PFW-30), 60 s (PFW-60) or 120 s (PFW-120). PFW was selected because it is widely used in the literature as a model material for wetting studies [23,24,25]. As shown in Figure 2a, when the PFW treatment was conducted for 30 s, the sample surface was partially coated with paraffin wax. Regions with a thick PFW layer were present around the edge of the cylindrical pores. It is likely that 30 s of dip-coating were not enough to completely wet the whole volume of the sample and thus only a partial coating was obtained. In addition, high magnification images of the pores revealed that the fibrous porosity of the fungus was not preserved by the treatment. The PFW-toluene solution penetrated in between the micro-fibres and, after toluene evaporation, PFW solidified and closed the pores. When the treatment time was increased to 60 and 120 s (Figure 2b,c, respectively), a thick PFW coating was created on the sample surface. The shape of pores was altered, and the micro-fibres were embedded inside PFW. In fact, the fibrous network was not visible for PFW-120 samples. For all cases, the internal volume of the micro-tubes remained free from PFW. This can be explained by considering the drying kinetics in porous capillaries [26,27,28]. As toluene evaporated from the PFW solution trapped inside the micro-tubes, the liquid–gas interface receded inside the tubes and a liquid meniscus was formed. The liquid at the tube walls started to solidify, leading to polymer accumulation near the drying interface and formation of a PFW film.

The presence of the PFW coating was also demonstrated by XRD analysis and wettability tests. The untreated fungi did not show a characteristic XRD pattern, due to their amorphous nature (Figure 2d). On the contrary, samples treated with PFW exhibited two obvious diffraction peaks at 21.5° and 24.0°, which correspond to [110] and [200] crystal planes of monoclinic paraffin, respectively [29,30]. As expected, the hydrophobic behaviour of the samples was enhanced by the PFW coating. WCA values in the range of 128°–135° were measured, PFW-120 being slightly less hydrophobic than PFW-30 (Figure 2e). This was due to a decrease in surface roughness with treatment time: PFW-120 surface was smoother than PFW-30 surface, because the micro-fibres were coated with PFW. However, no statistically significant differences between the samples were observed. Our results are in agreement with previous studies that have reported WCA values between 110° and 130° for PFW surfaces [31,32,33]. For example, Paul et al. used PFW solutions in toluene to treat filter paper and make it hydrophobic with a WCA of 126° [31]. They also showed that the treated paper had a high separation efficiency for oil-in-water non-stabilised emulsions. 

**Figure 3:**
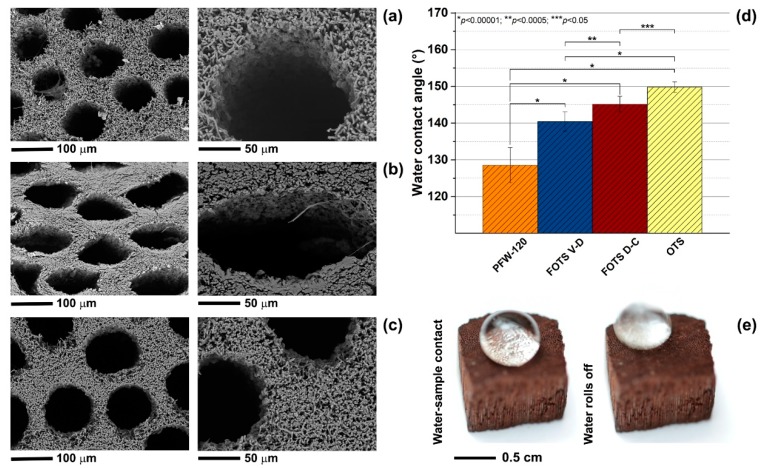
SEM images, with two different magnifications, of the surface porosity of *G. applanatum* samples treated with (**a**) FOTS by vapour deposition, (**b**) FOTS by dip-coating and (**c**) OTS by dip-coating. (**d**) Water contact angle values of the treated samples. (**e**) Photographs of one water drop impacting on the surface of the OTS-treated sample.

Although the PFW treatment allowed us to achieve hydrophobicity, the porosity of the fungus was modified, and only cylindrical pores were left open. For this reason, surface treatments based on small molecules (FOTS and OTS) were conducted. As shown in Figure 3a–c, FOTS and OTS treatments had no effect on the micro-porosity of the samples. The microfibres were still visible after the functionalisation procedures, without closure of the voids between them. It is expected that the FOTS and OTS molecules create a thin coating (few nm) around the fibres without modifying their morphology, as reported by previous studies on natural fibres [34,35,36]. After the FOTS and OTS functionalisation, no changes in the weight of the samples were registered. The geometry of the cylindrical pores was identical to that of the original fungus for the FOTS treatment performed by vapour deposition (FOTS V-D, Figure 3a) and for the OTS treatment (Figure 3c). On the contrary, the cross-section of the micro-tubes became elongated and deformed when the FOTS treatment was conducted by dip-coating (FOTS D-C, Figure 3b). In this case, the samples were kept in the FOTS/ethanol solution for 4 h to achieve a uniform coating of the samples (small variations of the surface wetting properties). This likely induced swelling of the samples and deformation during drying.

Wetting studies showed a significant increase in the water contact angle values for the silane-based coatings when compared with PFW (Figure 3d). WCA values between 140°–145° were measured for samples treated with FOTS, whereas WCA values higher than 150° were achieved for the OTS coating. The OTS-treated samples were superhydrophobic, with a low contact angle hysteresis. As shown in Figure 3e (left-hand side), when the water drop impacted onto the OTS-coated surface of the fungus (the distance between the droplet and the sample surface was 5 mm), air pockets were formed between the water drop and the features on the surface of the sample. Due to the dual-scale roughness of the fungus surface (porosity from few to hundred µm), the air was trapped inside the micro-tubes (with rough walls) and between the protruding hydrophobic microfibres. This wetting state can be described by the Cassie-Baxter model [37,38], where the composite contact area (solid and air pockets) is responsible for low contact angle hysteresis and low sliding angles [39,40,41,42]. In the case of the OTS-treated fungi, the water drop rolled off the sample surface immediately after impact (Figure 3e, right-hand side), with a contact angle hysteresis of about 2° (advancing WCA of 143°, receding WCA of 141°). The mechanical stability of the OTS coating was investigated after abrasion of the surfaces. The abraded samples maintained the hydrophobic character with WCA of 140°, but the water droplet did not roll off the sample surface anymore, indicating that the superhydrophobic or self-cleaning behaviour, was lost. 

**Figure 4:**
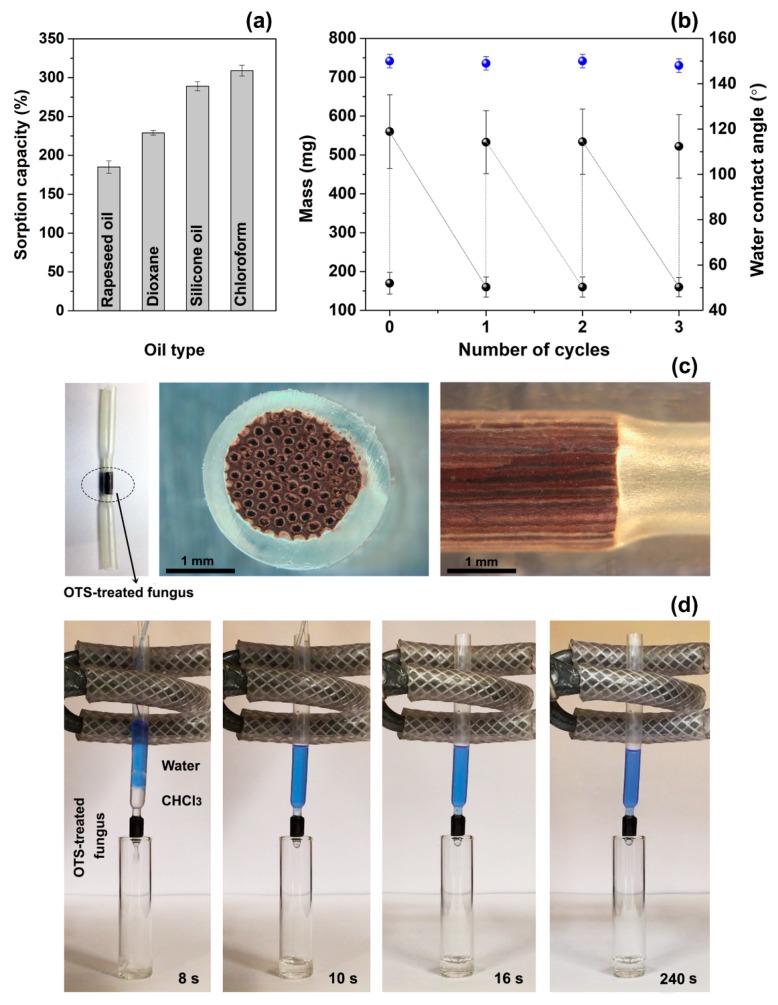
(**a**) Sorption capacity of OTS-treated samples for rapeseed oil, dioxane, silicone oil and chloroform. (**b**) Reusability of the OTS-treated fungus for dioxane: mass of dried and wet samples (black circles), and water contact angle (blue circles). (**c**) Photographs of the OTS-treated fungus inside a heat-shrinkable tube, showing that the cross section is not damaged or deformed. (**d**) Frames extracted from the video of the separation of water (blue liquid) from chloroform (transparent liquid). Water has not permeated through the OTS-treated fungus at the pressure difference of about 170 Pa.

Oil sorption tests showed that the OTS-functionalised samples were able to absorb oils and organic solvents with a sorption capacity of (185 ± 8)%, (229 ± 3)%, (289 ± 6)% and (309 ± 7)% for rapeseed oil, dioxane, silicone oil and chloroform, respectively (Figure 4a). These values are similar to those reported for other natural fibrous materials, such as loofah sponges modified with fluorinated silica nanoparticles (~300% for chloroform) [10], hydrophobic cotton fibres (~500% for vegetable oil) [43] and coir fibres (180–540% for crude oil) [44]. The possibility to recycle and reuse the OTS-treated samples was evaluated by sorption/purification cycles. The samples maintained their original super-hydrophobicity (WCA of 150°) and sorption capacity ((230 ± 4)% for dioxane) after four cycles of dioxane adsorption and ethanol washing (Figure 4b). No apparent deterioration of the structure of the fungus and functionality of the coating was observed, indicating that the samples can be recycled and used multiple times. 

The ability of the fungus to repel water while adsorbing oil, and its unique porosity were tested for oil-water separation. Cylindrical pieces, 3 mm in diameter, were cut out from the OTS-modified samples by means of a metal hallow punch. They were fixed into a heat-shrinkable transparent tube (supplied by Falcon Workshop Ltd., Wigan, UK) in order to have the fungus’ micro-capillaries parallel to the main axis of the tube (Figure 4c). The mechanical rigidity of the *G. applanatum* samples allowed the walls of the heat-shrinkable tube to perfectly adhere to the external surface of the fungus, without deformation of the cross-section of the micro-capillaries. Water and chloroform were simultaneously injected inside the tube, and the time needed for the sample to separate the two liquids was recorded. While water (blue liquid in Figure 4d) was stopped by the hydrophobicity of the fungus and remained inside the tube, chloroform (transparent liquid in Figure 4d) passed through the fungus at a rate of 0.1 mL/s and was collected inside the glass vial. It is worth noting, that the difference in density between the two liquids caused the spontaneous formation of a lighter layer (water) at the top of the tube and a heavier layer (chloroform) at the bottom (left panel in Figure 4d). The specific morphology of the fungus, including regular and long micro-capillaries in the direction of the liquid flow, combined with a high hydrophobicity level of the fungus surfaces, resulted in the creation of a highly selective semipermeable barrier which enabled the chloroform/water separation process. The ability to easily integrate a natural porous material, acting as a selective flow barrier, within a fluidic tubing has not been reported before. This new approach offers exciting opportunities for the rapid and low-cost fabrication of fluidic networks for liquid filtration and selective flow controls enabled by tubing-integrated chemically-modified fungi. 

## 4. Conclusions

In this study, we show that *Ganoderma* fungi can be considered a source of 3D biodegradable porous materials with low environmental impact for oil/water separation applications. These fungi are widely available as they are involved in the natural decomposition of dead wood. Here, we treated the fungi surface with silane-based self-assembled monolayers, which are widely used to make a variety of materials super-hydrophobic [45]. The fungi were used without further processing, but pyrolysis can be implemented in the future to reduce the initial weight of the fungi. We obtained oil sorption capacities that were lower than those reported for synthetic sponges and membranes [46,47,48], but comparable to those of natural, non-pyrolyzed fibrous materials. The chemical components of the fungi (glucans and chitin) are mainly water-insoluble and resistant to organic solvents and acid solutions [49,50,51], making these porous systems stable in harsh environments. This work opens the way to a novel use of *Ganoderma* fungi as filtration/purification elements that can also be integrated into fluidic devices and used to separate emulsified oil from water.
